# Differential Expression of Key Oncogenic and Tumor Suppressor MicroRNAs Induced by Andrographolide in Androgen-Independent PC3 and Androgen-Dependent LNCaP Prostate Cancer Cells

**DOI:** 10.3390/genes16121514

**Published:** 2025-12-17

**Authors:** Padmavati Sahare, Luis Alberto Bravo-Vázquez, Diego Antonio Veloz-Briones, Daniela Bernal-Vázquez, Ignacio Bolaños-Fernández, Brenda Anguiano, Gabriel Luna-Bárcenas, Sujay Paul

**Affiliations:** 1 Institute of Advanced Materials for Sustainable Manufacturing, Tecnologico de Monterrey, Campus Queretaro, Queretaro 76130, Mexico; padma.sahare@tec.mx (P.S.); gabriel.luna@tec.mx (G.L.-B.); 2 School of Engineering and Sciences, Tecnologico de Monterrey, Campus Queretaro, Queretaro 76130, Mexico; 3 Instituto de Neurobiología, Universidad Nacional Autonoma de Mexico, Campus UNAM 3001, Juriquilla 76230, Mexico; anguianoo@unam.mx

**Keywords:** andrographolide, prostate cancer, microRNA expression, cytotoxicity, PC3 and LNCaP cells

## Abstract

Background: Prostate cancer remains a major contributor to cancer-related morbidity and mortality worldwide, emphasizing the need for safer and more effective therapeutic options. Andrographolide, a diterpenoid lactone derived from *Andrographis paniculata*, has shown promising anticancer activity, yet its effects on microRNA (miRNA) regulation in prostate cancer remain insufficiently explored. Methods: In this study, we evaluated the cytotoxic and molecular effects of andrographolide on two human prostate cancer cell lines, PC3 and LNCaP, along with HEK-293 cells as a noncancerous model. Results: Cell viability assessment using the MTT assay revealed dose-dependent cytotoxicity, with 24 h IC_50_ values of 82.31 µM for PC3, 68.79 µM for LNCaP, and 133.9 µM for HEK-293 cells. Subsequent expression analysis of key oncogenic and tumor suppressor miRNAs demonstrated that andrographolide induced the upregulation of miR-16-5p, miR-34a-5p, and miR-200a-5p miRNAs implicated in apoptosis, proliferation control, and androgen receptor signaling. In contrast, the expression of oncomiRs miR-21-5p and miR-221-5p showed minimal or nonsignificant changes, reflecting the complex and context-specific roles of miRNAs in prostate cancer. Gene expression profiling further indicated differential transcriptional responses between the two prostate cancer cell lines, consistent with their distinct molecular backgrounds. Conclusions: Although HEK-293 cytotoxicity and previously reported nephrotoxic effects warrant caution, these results support the potential of andrographolide as an adjuvant phytochemical capable of modulating clinically relevant miRNAs in prostate cancer. Future studies investigating optimized delivery systems and validating direct miRNA targets may help advance andrographolide toward safer and more targeted therapeutic applications.

## 1. Introduction

Prostate cancer is the second most frequently diagnosed malignancy in men worldwide, with approximately 1.4 million new cases and 375,000 deaths reported in 2020, remaining the most detected cancer among men in over 100 countries [1]. Most prostate cancers are adenocarcinomas, meaning tumors that start in gland cells and usually develop in the outer region of the prostate, with wide variation in their behavior and genetic makeup [2]. Dysregulation of key pathways, such as Androgen Receptor (AR) signaling, phosphoinositide 3-kinase/protein kinase B/mammalian target of rapamycin (PI3K/AKT/mTOR) activation, and DNA repair defects, plays a central role in tumor initiation and resistance to therapy, particularly through Phosphatase and Tensin Homolog (PTEN) loss and persistent AR activity [3]. Epigenetic alterations, including abnormal DNA methylation and histone modifications, also contribute to prostate cancer progression by remodeling the tumor microenvironment and promoting metastasis-associated gene expression [4]. At the molecular level, mutations in genes such as breast cancer 1 (*BRCA1*), breast cancer 2 (*BRCA2*), *PTEN*, and homeobox B13 (*HOXB13*) have been strongly associated with prostate cancer development, disrupting key pathways related to DNA repair, tumor suppression, and androgen signaling, highlighting the importance of molecular profiling for both risk prediction and targeted therapy [5]. The inactivation of the tumor suppressor gene Tumor Protein 53 (*TP53*) represents a pivotal event in malignant progression, as mutation rates escalate from 8% in localized cancer to as high as 73% in metastatic castration-resistant prostate cancer, correlating with a significantly higher risk of disease recurrence and poor patient outcomes [6]. Upregulation of the anti-apoptotic protein B-Cell Lymphoma 2 (BCL-2), observed to increase with Gleason grade, a system used to evaluate the aggressiveness of prostate cancer based on microscopic appearance, and disease progression, confers a significant survival advantage to tumor cells and is a key mechanism for acquiring resistance to androgen-targeted therapies [7]. Conversely, the pro-apoptotic effector protein Bcl-2-associated X protein (BAX) is consistently expressed in 100% of prostate cancer samples across all disease stages, indicating that the core machinery for apoptosis remains intact but is actively antagonized by pro-survival proteins [8]. Aberrant activation of the Wingless-related integration site (Wnt) signaling pathway is a common feature of advanced prostate cancer, where it drives tumor progression, treatment resistance, and metastasis by controlling stem cell function, proliferation, and motility [9]. Gain-of-function mutations in the Catenin Beta 1 (*CTNNB1*) gene, which are more prevalent in metastatic disease (4.3–5.4%) than in primary tumors (1.8–2.6%), cause hyperactivation of the Wnt pathway by stabilizing the β-catenin protein, thereby promoting an aggressive clinical course and resistance to both chemotherapy and AR inhibitors [10].

To study these complex molecular mechanisms and therapeutic responses, prostate cancer cell lines such as LNCaP and PC3 are widely used in vitro models, representing distinct disease stages and phenotypes. LNCaP originates from a lymph node metastasis of prostate cancer and retains an aneuploid state, exhibiting abnormal chromosomal counts that confer high androgen sensitivity and robust AR expression. Typically, prostate cancer cells depend on androgen receptor signaling to regulate differentiation and disease progression. Functionally, LNCaP cells produce prostate-specific antigen (PSA) and NKX3.1, both of which are markers of prostate epithelial differentiation and key targets of AR signaling. Moreover, LNCaP cells are wild-type for TP53, maintaining an intact apoptotic pathway, which makes them particularly valuable for studying androgen-dependent mechanisms of prostate cancer [11,12]. On the other hand, PC3 cells represent one of the most aggressive and extensively studied prostate cancer cell lines, derived from bone metastasis of a grade IV prostatic adenocarcinoma. Compared with LNCaP and other established prostate cancer cell lines, PC3 cells exhibit greater invasive capacity and adaptability to varying microenvironmental conditions. The mechanical properties of PC3 cells, such as stiffness, contractility, and motility, are strongly influenced by the rigidity of their surrounding substrate, reflecting the mechanical characteristics of the bone tissue from which they were isolated [13,14].

Despite the availability of conventional treatments including surgery, radiation, and chemotherapy, advanced prostate cancer frequently becomes resistant within two years, and these therapies are often associated with significant side effects such as urinary incontinence, erectile dysfunction, bowel dysfunction, and fatigue, which further diminish patients’ quality of life [15,16].

**Figure 1:**
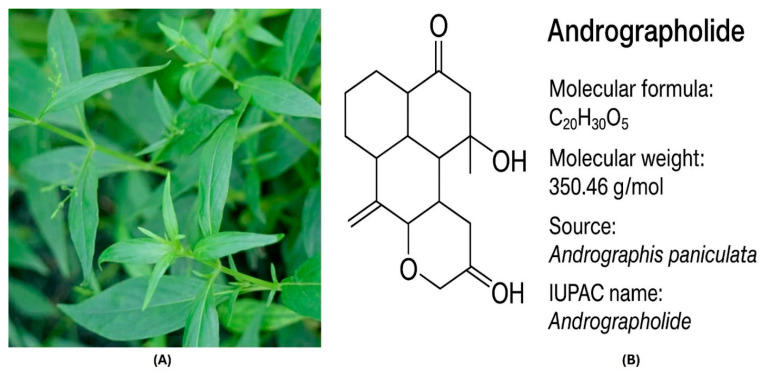
*A. paniculata* (**A**) and the chemical structure (**B**) of its major bioactive constituent, andrographolide.

Andrographolide is a labdane diterpene lactone isolated from the medicinal plant *A. paniculata* (Burm. f.) Nees (Figure 1A), commonly known as the “King of Bitters”, has long been used in traditional Asian medicine for its antipyretic, anti-inflammatory, and hepatoprotective activities [17]. Structurally, andrographolide possesses a decalin ring system with a γ-lactone moiety and multiple hydroxyl groups (Figure 1B), conferring moderate lipophilicity and limited aqueous solubility, which in turn restricts its bioavailability and therapeutic translation [18,19]. Despite these pharmacokinetic challenges, andrographolide has demonstrated potent anticancer effects across several malignancies, including prostate, breast, colon, and pancreatic cancers, primarily through modulation of critical signaling pathways that regulate cell survival, proliferation, and apoptosis [20]. Mechanistically, it suppresses constitutive activation of nuclear factor kappa B (NF-κB) and Janus kinase/signal transducer and activator of transcription 3 (JAK/STAT3) signaling, thereby inhibiting tumor-promoting inflammation and enhancing apoptotic signaling [21]. In prostate cancer models, andrographolide reduces androgen receptor (AR) expression and PI3K/Akt/mTOR pathway activity, promoting G2/M phase arrest and intrinsic apoptosis through upregulation of pro-apoptotic proteins such as Bax, caspase-3, and caspase-9, and concurrent downregulation of anti-apoptotic Bcl-2 [22,23]. Furthermore, it induces reactive oxygen species (ROS) accumulation and mitochondrial membrane depolarization, amplifying apoptotic sensitivity in androgen-independent prostate cancer cells [24]. Beyond its direct cytotoxicity, andrographolide has been shown to attenuate epithelial–mesenchymal transition (EMT), migration, and invasion by inhibiting transforming growth factor-beta (TGF-β) and Wnt/β-catenin signaling axes in alveolar epithelial cells [25]. Its ability to target cancer stem cell populations and enhance chemosensitivity, particularly through modulation of multidrug resistance transporters, further underscores its potential as a natural adjunct in prostate cancer therapy.

MicroRNAs (miRNAs) are a class of small, non-coding RNAs that post-transcriptionally regulate gene expression, and their altered expression is known to be associated with the establishment and progression of various human cancers [26,27,28]. In the canonical pathway, miRNA biogenesis begins with the transcription of primary miRNAs (pri-miRNAs) by RNA polymerase II. This is followed by sequential processing by the microprocessor complex (Drosha-DGCR8) in the nucleus and Dicer in the cytoplasm to generate mature miRNAs capable of guiding the RNA-induced silencing complex (RISC) to their target mRNAs [29]. This maturation process is further fine-tuned by RNA-binding proteins that recognize specific sequences or structural motifs in pri- or pre-miRNAs, influencing their processing efficiency and ensuring precise regulation of miRNA abundance and function [30]. In prostate cancer, dysregulation of miRNAs is evident in the oncogenic overexpression of miR-21, which drives tumor progression, and the loss of tumor suppressor clusters such as miR-15a/16-1, leading to impaired control of cell proliferation and survival [31]. This illustrates the dual functionality of miRNAs, which can act either as tumor suppressors, such as miR-34a in prostate cancer, or as oncogenes (onco-miRs), making them powerful candidates for the development of clinical biomarkers for cancer diagnosis, prognosis, and therapeutic response [32]. Moreover, in prostate cancer, miR-34a is markedly underexpressed in cancer stem cell (CD44^+^) subpopulations; enforced re-expression of miR-34a significantly inhibits tumor regeneration and metastasis, likely through targeting of stemness and survival regulators such as CD44, NOTCH, MYC, and BCL-2 [33]. The miR-200 family acts as tumor suppressor miRNAs in prostate cancer with effects on EMT, metastasis, and cell invasion [34]. Interestingly, MiR-221/222 was highly expressed in bone metastatic CRPC tumor specimens. Overexpressing miR-221 in LNCaP reduced androgen-dependent growth arrest and activated epithelial-to-mesenchymal-transition (EMT) and metastasis-related genes (HECTD2 and RAB1A identified as targets) [35]. However, another study found that miR-221-5p is significantly downregulated in prostate cancer vs. benign tissue and lower in metastasis compared to primary prostate cancer; yet when overexpressed, it reduced proliferation and migration [36]. This suggests a context-dependent role of microRNAs.

Although some of the potential molecular mechanisms underlying the anticancer effects of andrographolide have been reported, its specific impact on miRNA expression profiles in prostate cancer cells is still elusive. Therefore, this study aims to investigate how andrographolide modulates key oncogenic and tumor suppressor miRNAs in PC3 and LNCaP cell lines, thereby uncovering a novel layer of its potential therapeutic mechanism.

## 2. Materials and Methods

### 2.1. Materials

Andrographolide (Purity: >99%, MCE, Monmouth Junction, NJ, USA), Dulbecco’s Modified Eagle Medium (DMEM, Gibco/Thermo Fisher Scientific, Waltham, MA, USA), Roswell Park Memorial Institute Medium (RPMI, Gibco/Thermo Fisher Scientific, Waltham, MA, USA), Fetal Bovine Serum (FBS, Gibco/Thermo Fisher Scientific, Waltham, MA, USA), PC3, LNCaP and HEK-293 (ATCC, Manassas, VA, USA). 3-(4,5-dimethylthiazol-2-yl)-2,5-diphenyltetrazolium bromide (MTT, Sigma-Aldrich, St. Louis, MO, USA), Oligos (CTR Scientific, Monterrey, Mexico), miRNeasy Mini Kit (Qiagen, Hilden, Germany), Mir-X miRNA First-Strand Synthesis Kit (Takara Bio Inc., Shiga, Japan), and Mir-X miRNA TB Green qRT-PCR kit (Takara Bio Inc., Shiga, Japan).

### 2.2. Cell Culture

In this study, two primary human prostate cancer cell lines were used: PC3, an aggressive metastatic androgen-independent adenocarcinoma line, and LNCaP, a metastatic androgen-dependent prostate carcinoma line. In addition, the human embryonic kidney cell line HEK-293 was included as a noncancerous control. PC3 and HEK-293 cells were cultured in DMEM supplemented with 10% FBS and 1% antibiotic–antimycotic solution. LNCaP cells were maintained in RPMI supplemented with 10% FBS and 1% antibiotic–antimycotic solution. All cell cultures were maintained at 37 °C in a humidified incubator with 5% CO_2_.

### 2.3. Cell Viability Assay

Cell viability was evaluated following andrographolide treatment, with untreated cells serving as the control. PC3 and LNCaP cells were seeded into 96-well plates at a density of 5000 cells per well and incubated for 24 h in their respective culture media. Andrographolide has limited aqueous solubility, a characteristic that presents challenges in experimental settings. To avoid introducing solvents that could interfere with the miRNA analyses, the compound (MCE, purity > 99%) was dissolved directly in complete culture medium (DMEM or RPMI). Fresh stock solutions were prepared at 1 mg/mL, vortexed thoroughly, and equilibrated at 37 °C to enhance dissolution. Working solutions were obtained by diluting the stock directly in culture medium, and 200 μL of each final concentration (25, 50, 75, 100, 125, and 150 μM) was added to the wells. After 24 h of treatment, the medium was removed, and the wells were washed with 200 μL of 1× PBS. Subsequently, 0.5 mg/mL of MTT solution was added to each well, and the plates were incubated for 3 h. The supernatant was then carefully removed, and the resulting formazan crystals were dissolved in 200 μL of dimethyl sulfoxide (DMSO). Absorbance was measured at 570 nm using a Multiskan SkyHigh microplate reader (Thermo Fisher Scientific, Waltham, MA, USA). In addition, cell viability was assessed in Human Embryonic Kidney (HEK-293) cells treated with andrographolide at concentrations ranging from 25 to 150 μM to evaluate its effect on normal, noncancerous cells. The experimental procedures were identical to those used for the prostate cancer cell lines. Cell viability was calculated as the percentage of absorbance in treated cells relative to untreated controls. The half-maximal inhibitory concentration (IC_50_) was defined as the concentration of andrographolide required to reduce cell viability by 50%. All treatments were performed in triplicate.

The following formula calculates cell viability:

(1) $$Cell\;viability\;\%=\;\left[\frac{OD\;treated\;cells}{OD\;control\;cells}\right]\times 100$$

### 2.4. RNA Extractions and cDNA Synthesis

PC3 and LNCaP cells were seeded into T-25 flasks. When the cells reached approximately 80% confluence, they were treated with andrographolide at its IC_25_ concentration. Treatments were carried out for 24 h, with untreated cells serving as the control group. Following incubation, total RNA was extracted and purified using the miRNeasy Mini Kit (Qiagen, Hilden, Germany) according to the manufacturer’s protocol. RNA concentration and purity were determined using a NanoDrop One spectrophotometer (Thermo Fisher Scientific, MA, USA). Prior to qPCR analysis, 1 μg of total RNA (including small RNAs) extracted from control and andrographolide-treated PC3 and LNCaP cells was polyadenylated and reverse-transcribed into cDNA using the Mir-X miRNA First-Strand Synthesis Kit (Takara Bio Inc., Shiga, Japan). cDNA synthesis was performed at 37 °C for 1 h, followed by enzyme inactivation at 85 °C for 5 min.

### 2.5. Experimental Validation and Expression Analysis of miRNAs and Target Genes by RT-qPCR

qPCR assays were carried out to evaluate the expression of five miRNAs (two oncomiRs and three tumor suppressor miRNAs), two cancer-associated target genes, and one inflammation-related gene. Regarding the primer sequences of miRNAs, the full-length sequence of each miRNA served as the forward primer, whereas the reverse primer corresponded to the mRQ 3′ primer (proprietary) supplied in the Mir-X miRNA First-Strand Synthesis Kit (Takara Bio Inc., Shiga, Japan). All the primer sequences are listed in Table 1. Each qPCR experiment was performed in a Step One Real-Time PCR System (Applied Biosystems, Carlsbad, CA, USA) with the reagents of the Mir-X miRNA qRT-PCR SYBR Kit (Takara Bio Inc., Shiga, Japan). The reaction mix consisted of 1× SYBR Advantage Premix, 1× ROX dye, forward and reverse primers (0.3 µM each one), and 1 µL of the samples of cDNA; the final volume was 12.5 μL. The qPCR experiments were carried out under the following conditions: initial denaturation at 95 °C for 10 s, followed by 45 amplification cycles consisting of denaturation at 95 °C for 5 s, annealing at 55 °C for 20 s, and extension at 72 °C for 20 s. The process concluded with a melting curve analysis of 95 °C for 1 min, 55 °C for 30 s, and 95 °C for 30 s. These qPCR reactions were performed with three biological replicates and two technical replicates corresponding to the samples of both control and treated cell lines. Lastly, the relative fold changes in miRNA and gene expression levels were determined using the comparative threshold cycle approach (2^−ΔΔCT^), commonly known as the delta–delta Ct method, with the small nuclear RNA U6 serving as the endogenous reference for normalization.

### 2.6. Statistical Analysis

The IC_50_ values were calculated using nonlinear regression (Inhibitor vs. normalized response) in GraphPad Prism (Version 10.4.2), incorporating all biological replicates. Results from at least three independent experiments are presented as the mean ± standard deviation or standard error. Statistical comparisons were performed using either a two-tailed Student’s *t*-test or ANOVA with Tukey’s post hoc test. Differences were considered statistically significant at * *p* < 0.05, ** *p* < 0.01, and *** *p* < 0.001.

## 3. Results

### 3.1. Cell Viability of PC3 and LNCaP Cells Following Andrographolide Treatment and Determination of IC50

**Figure 2:**
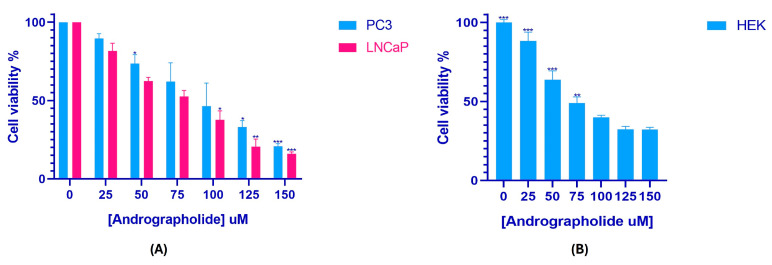
Andrographolide reduces cell viability in a concentration-dependent manner. MTT assay showing the effect of andrographolide (0–150 µM, 24 h) on the viability of PC3, LNCaP (**A**), and HEK-293 cells (**B**). IC_50_ values were calculated from the corresponding dose–response curves. Values represent mean ± SD from at least three independent experiments. Differences were considered statistically significant at * *p* < 0.05, ** *p* < 0.01, and *** *p* < 0.001.

**Figure 3:**
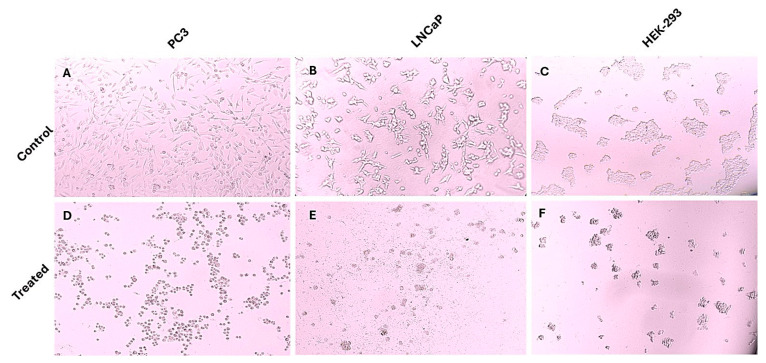
Morphological changes in PC3, LNCaP, and HEK-293 cells following andrographolide treatment. Representative microscopic images of untreated control cells and cells treated with the highest concentration of andrographolide used in this study (150 µM). Andrographolide exposure induced noticeable morphological alterations in all three cell types, including cell shrinkage, loss of adherence, and reduced cell density, consistent with cytotoxic effects. (**A**) PC3 untreated control; (**B**) LNCaP untreated control; (**C**) HEK-293 untreated control; (**D**) PC3 treated; (**E**) LNCaP treated; (**F**) HEK-293 treated.

This study included a cell viability assay using the MTT method to determine the impact of andrographolide on prostate cancer cell proliferation and HEK-293 cell viability across a concentration range of 0–150 µM over a 24 h period. The control group consisted of cells that were left untreated. As illustrated in Figure 2, andrographolide treatment resulted in a concentration-dependent reduction in the viability of PC3, LNCaP, and HEK-293 cells. Based on these results, the IC_50_ values were determined to be 82.31 µM for PC3, 68.79 µM for LNCaP, and 133.9 µM for HEK-293. Additionally, microscopic images of treated and untreated cells (Figure 3) show clear morphological alterations following andrographolide exposure, including reduced cell density, cell shrinkage, and loss of typical morphology in PC3 and LNCaP cells, further supporting the observed decrease in cell viability.

### 3.2. Analysis of miRNA and Gene Expression Following Andrographolide Treatment

**Figure 4:**
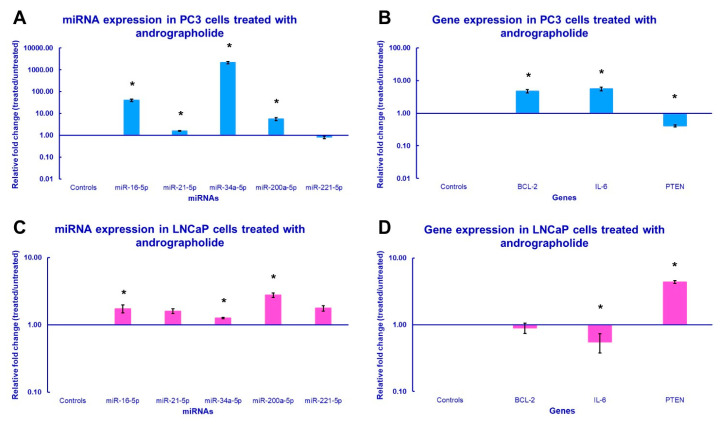
miRNA and gene expression profile of the prostate cancer cells treated with andrographolide. The qPCR analysis of (**A**,**C**) miRNA expression and (**B**,**D**) gene expression was conducted using total RNA extracted from PC3 and LNCaP cells that were subjected to the IC25, 41.16 µM and 34.4 µM, respectively, of andrographolide for 24 h. The bars in the graph indicate the average relative fold change and error bars represent the standard error derived from the biological replicates (* *p*-value < 0.05).

To investigate the regulatory effects of andrographolide on miRNA and gene expression, PC3 and LNCaP cells were incubated for 24 h with andrographolide at their respective IC_25_ concentrations (41.16 µM and 34.4 µM). The analysis focused on profiling the expression of miR-16-5p, miR-21-5p, miR-34a-5p, miR-200a-5p, miR-221-5p, *IL-6*, *BCL-2* and *PTEN* for both cell lines, with expression levels compared against untreated controls. According to the results of the qPCR assays, miR-16-5p, miR-21-5p, miR-34a-5p, and miR-200a-5p were significantly upregulated in the PC3 cell line after treatment, while miR-221-5p was downregulated in the same cells, but the result did not statistically differ from the control cells (Figure 4A). On the other hand, almost all miRNAs were significantly upregulated in the LNCaP cell line, with the exception of miR-21-5p and miR-221-5p, which did not show statistical significance for claiming their upregulation when compared to the control groups (Figure 4C). Regarding the expression levels of the genes of interest, both *IL-6* and *BCL-2* were significantly upregulated in PC3 cells, and *PTEN* was significantly downregulated (Figure 4B). Conversely, *IL-6* was significantly downregulated in LNCaP cells, while *PTEN* was significantly upregulated. The downregulation of *BCL-2* in this cell line was not statistically significant (Figure 4D).

## 4. Discussion

Prostate cancer remains one of the leading causes of cancer-related morbidity and mortality among men worldwide, underscoring the urgent need for safer and more effective therapeutic strategies. In this context, plant-derived bioactive compounds have gained considerable attention for their potential anticancer properties [37,38,39,40]. Andrographolide, the major diterpenoid lactone isolated from *A. paniculata*, has emerged as a promising natural candidate for prostate cancer therapy [41,42]. Several studies have demonstrated that andrographolide can suppress prostate cancer cell proliferation, induce apoptosis, and modulate key signaling pathways such as NF-κB, PI3K/Akt, and MAPK in prostate cancer models. However, its specific impact on miRNA expression profiles in prostate cancer cells remains unclear. In our study, the IC_50_ values for andrographolide after 24 h were 82.31 µM for PC3 and 68.79 µM for LNCaP cells. These results corroborate those of Manimaran and Azman [43], who reported an LC_50_ of 64.98 ± 9.51 μM for PC3 cells after 24 h (The LC_50_ values reported by the authors are numerically similar to the IC_50_ values obtained in our study, suggesting consistent cytotoxicity profiles despite the different endpoints measured). The minor differences may stem from the compound’s low hydrophilicity; since no solvent was used to dissolve andrographolide in our experiments, its bioavailability in the culture medium may have been slightly reduced. Furthermore, prolonged incubation enhances andrographolide’s cytotoxicity, with an IC_50_ of 26.42 ± 1.52 μM reported after 48 h. This increased potency may relate to the accumulation of reactive oxygen species (ROS). As noted by Iqbal et al. [44], sustained ROS elevation disrupts cellular homeostasis, damages mitochondria, and activates apoptotic pathways. Cancer cells, which inherently maintain higher baseline ROS levels, are particularly susceptible to oxidative stress, whereas normal cells possess stronger antioxidant defenses, providing greater resistance to ROS-induced damage.

For the LNCaP cell line, the IC_50_ value obtained in this study aligns with the findings of Mir et al. [41], who reported an IC_50_ of approximately 50 µM after 48 h of treatment. The authors also observed that andrographolide did not significantly reduce viability in normal prostate cells. In our work, the inhibitory effect of andrographolide was further evaluated in HEK-293 cells, yielding an IC_50_ of 133 μM, suggesting that healthy cells may also be affected at higher concentrations. Although HEK-293 cells are frequently used as a noncancerous (healthy) model, their transformed phenotype makes them more responsive to cytotoxic compounds than primary cells. Therefore, the comparable IC_50_ values observed here may reflect the intrinsic sensitivity of HEK-293 cells rather than a lack of selectivity of andrographolide. This is consistent with prior studies reporting moderate selectivity of andrographolide across transformed cell lines. Previous studies have shown that andrographolide can exert nephrotoxic effects, inducing inflammation and endoplasmic reticulum stress in HK-2 cells, and that it tends to accumulate in kidney tissue at concentrations of approximately 150 µg/g [45,46]. It is also important to note that andrographolide has been associated with adverse effects in both in vivo studies and clinical trials. Nonetheless, these side effects generally occur at high doses (>150 mg), particularly with frequent or long-term use, likely due to tissue accumulation [45]. Therefore, employing an appropriate delivery system and targeted therapeutic approach is crucial to maximize the anticancer potential of this potent phytochemical while minimizing off-target effects and systemic toxicity.

Having established the differential cytotoxic effects of andrographolide across prostate cancer and normal cells, we then explored its impact at the molecular level, particularly on oncogenic and tumor suppressor microRNAs. In this context, the observed upregulation of miR-16-5p in both cell lines aligns with its known function as a tumor suppressor miRNA. The upregulation of miR-16-5p in both cell lines is congruent with its role as a tumor suppressor miRNA. In fact, this miRNA was previously found to be downregulated in the PC3, LNCaP, and DU145 cell lines. Furthermore, a treatment with a miR-16-5p mimic demonstrated that this miRNA could induce apoptosis and reduce the viability of prostate cancer cells by targeting a key target gene associated with carcinogenesis, i.e., AKT3 [47]. Similar results were observed by Jin et al. [48] after inducing the overexpression of the miR-15a/16 cluster in LNCaP cells, thereby suppressing the TGF-β signaling pathway and reducing LNCaP invasion. In this context, our results imply that andrographolide treatment induces miR-16-5p expression in PC3 and LNCaP cells, potentially promoting cell death through the regulation of AKT3 and TGF-β signaling.

In the case of miR-34a-5p, it has been shown that the expression of this miRNA is downregulated in prostate cancer cases carrying *TP53* deletions or mutations. Moreover, introducing a synthetic miR-34a mimic into PC3 cells resulted in the suppression of several miR-34a downstream targets (e.g., CD44, Cyclin D1, and BCL-2) and a marked reduction in cellular proliferation [49]. Also, a recent study indicated that miR-34a-5p expression became upregulated in PC3 cells after a treatment with green tea extract, reducing cell viability via targeting several proteins involved in the regulation of cell proliferation, such as cyclin B1, c-Myc, and p53 [50]. Accordingly, the overexpression of miR-34a-5p induced by andrographolide in our study may also have contributed to the reduction in PC3 and LNCaP growth by targeting a number of genes that promote cancer cell proliferation. Furthermore, it is worth noting that the anticancer effect observed in our study may not be directly associated with the downregulation of *BCL-2*, as this result was not observed in either cell line. This highlights the importance of future research analyzing how andrographolide treatment affects the expression of other direct targets of miR-34a-5p.

On the other hand, Guan et al. [51] discovered that the expression levels of miR-200a-5p were markedly downregulated in tumor samples obtained from individuals with castration-resistant prostate cancer. Further statistical evaluation identified miR-200a-5p as an independent prognostic indicator associated with patient outcomes in prostate cancer. These authors also evidenced that the overexpression of miR-200a-5p diminished proliferation and promoted apoptosis in C4-2B and LNCaP cell lines through the suppression of the BRD4-mediated androgen receptor signaling pathway. Therefore, the upregulation of miR-200a-5p promoted by the andrographolide treatment might impede the aforementioned pathway in PC3 and LNCaP cells and suppress cell proliferation. Nevertheless, additional analyses are required to validate whether BRD4 becomes downregulated in these cell lines after the treatment with the phytochemical.

Due to their function as oncomiRs, miR-21-5p and miR-221-5p were expected to be downregulated following andrographolide treatment. In this matter, these two miRNAs represent promising biomarkers for prostate cancer diagnosis, given that they have been found to be significantly upregulated in prostate cancer tissues when compared to normal tissues [52]. In addition, both in vitro and in vivo findings revealed that the deletion of miR-21 inhibits prostate tumor development by disrupting the IRS1/SREBP-1 signaling cascade [53]. In addition, Ashrafi Dehkordi et al. [54] reported that the suppression of miR-221 expression markedly reduces the viability of prostate cancer cells by upregulating the p27 gene and promoting apoptosis. However, in PC3 cells, miR-21-5p expression increased slightly after treatment with the phytochemical, while the change in miR-221-5p expression was not statistically significant. Similarly, the changes in the expression of both oncomiRs in LNCaP cells were not statistically significant.

The above can be explained by the fact that the diverse and sometimes contradicting functions of miRNAs across tumor compartments and cell types complicate their therapeutic application [55]. Evidence from the NCI-60 cancer cell line panel showed that the same miRNA can mediate contrasting effects of a particular anticancer drug depending on the tumor type and miRNA levels in the cell. Remarkably, artificially induced fluctuations (through pre-miRNA or antisense oligomer transfection) in miR-21 levels were found to alter efficacy in contrasting ways across different classes of anticancer compounds, suggesting that distinct underlying mechanisms govern the toxic and protective roles of these therapeutic agents [56]. Likewise, certain miRNAs display both pro- and anti-angiogenic activities, variably influencing drug efficacy within the same cells [55]. Hence, it is probable that the anticancer effects of andrographolide are not associated with the functions of miR-21-5p and miR-221-5p, although that does not rule out the possibility that other oncomiRs have become downregulated after the treatment.

It is worth noting that the generalized upregulation in miRNA expression observed may partially reflect non-specific responses associated with cytotoxic stress, such as altered miRNA processing, apoptosis-associated RNA release, or global shifts in transcriptional stability [57]. This fact implies that the increased expression of certain RNA species (like miRNAs and cancer-associated transcripts) is indeed consistent with global transcriptional stress responses often observed during apoptosis. However, the expression of the U6 snRNA remained unchanged between treated and control groups, indicating the absence of RNA degradation and supporting the reliability of U6 snRNA as a normalization control under these conditions. On the other hand, regarding the panel of prostate cancer cells considered in our expression analyses, HEK293 cells were not included in qPCR expression analyses because the study was designed to compare androgen-dependent (LNCaP) and androgen-independent (PC3) prostate cancer cell types. Furthermore, HEK293 cells display highly variable basal miRNA levels [58], which may mask cancer-specific regulatory patterns. Considering this, it would be very convenient for further studies analyzing miRNA expression in treated prostate cancer cells to include normal prostate epithelial cells (e.g., HPrEC) as a control to validate miRNA expression under both basal and cytotoxic conditions.

Baseline differences in gene expression between PC3 and LNCaP prostate cancer cell lines can also influence their transcriptional responses following exposure to an anticancer agent such as andrographolide. As a matter of fact, Dozmorov et al. [59] reported that 2,198 genes were differentially expressed between PC3 and LNCaP cells. Consistently, it is important to consider the differences in baseline gene expression among different prostate cancer cell lines during experimental studies [60]. For instance, Gano et al. [61] found that the anticancer efficacy of synergistic phytochemical treatments is affected by the genetic background of prostate cancer cell lines. In this study, the genes analyzed exhibited opposite expression patterns after treatment, likely reflecting the intrinsic molecular characteristics of each cell line.

Notably, PC3 cells, the more aggressive prostate cancer model, retained an expression profile consistent with their malignant phenotype, characterized by *BCL-2* overexpression, *PTEN* downregulation, and increased expression of the inflammation-related gene *IL-6*. This may imply that the anticancer effects of the phytochemical applied herein are related to the downregulation of other oncogenes and the upregulation of other tumor suppressors in PC3. This, in turn, may also be related to the fact that a higher concentration of andrographolide was required to achieve a cytotoxic effect on PC3. Notwithstanding this, the upregulation of *PTEN* in LNCaP cells is consistent with the findings of an investigation in which the same cell line was treated with eupatilin (a phytochemical belonging to a secondary metabolite of *Artemisia asiatica*), which induced the upregulation of the tumor suppressor PTEN and prevented cell proliferation [62]. In relation to the miRNA patterns observed, the downregulation of *PTEN* transcripts in PC3 cells corresponds with the slight increase in miR-21-5p, a well-known direct repressor of PTEN, whereas the upregulation of *PTEN* in LNCaP cells aligns with the absence of significant changes in miR-21-5p, suggesting reduced miRNA-mediated repression in this line. Similarly, although *BCL-2* transcripts increased in PC3 cells, this finding is compatible with the concomitant upregulation of miR-16-5p and miR-34a-5p, both recognized inhibitors of *BCL-2* translation, indicating that BCL-2 protein levels may nevertheless be reduced despite elevated mRNA. In LNCaP cells, where *BCL-2* transcription did not change significantly, the strong induction of these tumor suppressor miRNAs likewise suggests decreased anti-apoptotic BCL-2 activity at the protein level. Therefore, andrographolide may promote the overexpression of this gene in LNCaP cells through mechanisms independent of the downregulation of miR-21-5p, which is one of the key miRNAs that target PTEN.

Regarding the anti-inflammatory activity of andrographolide in prostate cancer cells, it has been demonstrated that *IL-6* levels are frequently elevated in the serum of many men with advanced, hormone-refractory prostate cancer [63]. Moreover, the upregulation of this proinflammatory cytokine has been previously reported following treatments such as docetaxel administration [64]. This suggests that in certain cell populations, such as the aggressive model of PC3 cells, *IL-6* overexpression may act as a resistance mechanism against anticancer agents. In contrast, this response was not observed in LNCaP cells, indicating that this line may be more susceptible to andrographolide treatment, as evidenced by the downregulation of *IL-6* expression following phytochemical exposure. In fact, Méndez-Clemente et al. [65] noticed that the simultaneous inhibition of STAT-3 and IL-6R via the application of stattic and tocilizumab suppresses prostate cancer cell proliferation, migration, and invasion by disrupting the IL-6/IL-6R/STAT-3 signaling pathway. The divergent *IL-6* responses observed here further reflect the influence of the miRNA-regulated PTEN and BCL-2 pathways, as *IL-6* upregulation in PC3 aligns with a more aggressive, PTEN-deficient context, whereas *IL-6* downregulation in LNCaP is consistent with enhanced tumor-suppressive signaling driven by their miRNA profile. This information suggests that the disruption of the IL-6/IL-6R/STAT-3 could also be associated with the mechanism of action of andrographolide in LNCaP cells.

We acknowledge that a key limitation of our study is that gene expression was assessed exclusively at the transcript level using qPCR, providing only an initial overview of how andrographolide may influence the expression of various oncogenic and tumor suppressor transcripts. Accordingly, these observations will require further validation through complementary approaches capable of examining global miRNA and/or mRNA changes in response to andrographolide treatment in prostate cancer cells, such as small RNA sequencing and total RNA sequencing. Likewise, Western blot analyses of the genes evaluated here will be essential to confirm our qPCR findings at the protein level. Finally, including proteins associated with apoptosis (e.g., caspase-3, AIF, and p21) within the expression analysis could greatly complement the results of this study by helping to reveal how these apoptosis-related markers are affected after the treatment with andrographolide.

In addition to these technical limitations, an important constraint of the present work is the absence of functional validation experiments to determine whether the miRNA changes observed are causally involved in the cytotoxic effects of andrographolide. Although our data demonstrate clear modulation of several tumor suppressor and oncogenic miRNAs, we did not perform gain- or loss-of-function assays (such as miRNA mimic or inhibitor transfection), target gene rescue studies, or pathway-specific functional tests. As a result, the regulatory roles of miR-16-5p, miR-34a-5p, miR-200a-5p, and the evaluated oncomiRs remain associative rather than mechanistic. Future studies incorporating functional assays, along with confirmation of downstream protein-level effects and the interrogation of direct miRNA–target interactions, will be essential to clarify the biological significance of these regulatory changes and to define the precise mechanisms by which andrographolide influences prostate cancer cell behavior.

## 5. Conclusions

Our findings demonstrate that andrographolide exerts notable anticancer activity against prostate cancer cells by reducing cell viability and modulating key tumor-associated microRNAs. The phytochemical selectively upregulated tumor suppressor miRNAs, including miR-16-5p, miR-34a-5p, and miR-200a-5p in PC3 and LNCaP cells, suggesting potential involvement of pathways such as AKT3, TGF-β, and BRD4-mediated androgen receptor signaling in its mechanism of action. Although the expected downregulation of the oncomiRs miR-21-5p and miR-221-5p was not consistently observed, this outcome likely reflects the complex, cell type-specific regulatory dynamics that characterize miRNA biology in prostate cancer. Differences in basal gene expression between PC3 and LNCaP cells further emphasize the importance of cellular context in shaping transcriptional responses to andrographolide. Importantly, the cytotoxicity detected in HEK-293 cells and previously reported nephrotoxic effects highlight the need for targeted delivery strategies to enhance therapeutic selectivity and minimize off-target toxicity. Overall, this study provides new evidence that andrographolide influences the expression of clinically relevant miRNAs in prostate cancer cells, supporting its potential as a complementary anticancer agent. Future work should focus on validating the direct molecular targets of the regulated miRNAs and evaluating optimized delivery systems to improve the safety and efficacy of andrographolide-based therapies.

## Figures and Tables

**Table 1:** List of primers used in the qPCR experiments.

miRNA/Gene Name	Associated Role	Forward Primer (5′-3′)	Reverse Primer (5′-3′)
miR-16-5p	Tumor suppressor	TAGCAGCACGTAAATATTGGCG (22 nt)	mRQ 3′ primer (proprietary sequence)
miR-21-5p	OncomiR	TAGCTTATCAGACTGATGTTGA (22 nt)	mRQ 3′ primer (proprietary sequence)
miR-34a-5p	Tumor suppressor	TGGCAGTGTCTTAGCTGGTTGT (22 nt)	mRQ 3′ primer (proprietary sequence)
miR-200a-5p	Tumor suppressor	CATCTTACCGGACAGTGCTGGA (22 nt)	mRQ 3′ primer (proprietary sequence)
miR-221-5p	OncomiR	ACCTGGCATACAATGTAGATTT (22 nt)	mRQ 3′ primer (proprietary sequence)
*BCL-2*	Oncogenic	GATGGGATCGTTGCCTTATGC	CTTGGCATGAGATGCAGGA
*PTEN*	Tumor suppressor	AGTCAGAGGCGCTATGTGT	CGTGTGGGTCCTGAATTGGA
*IL-6*	Inflammation-related	ACTCACCTCTTCAGAACGAATTG	CCATCTTTGGAAGGTTCAGGTTG
*U6*	Endogenous control	GGAACGATACAGAGAAGATTAGC	TGGAACGCTTCACGAATTTGCG

## Data Availability

The data that support the findings of this study are available from the corresponding author upon reasonable request.

## Abbreviations

AKT	Protein Kinase B
AR	Androgen Receptor
BCL-2	B-Cell Lymphoma 2
BRCA1	Breast Cancer 1 Gene
BRCA2	Breast Cancer 2 Gene
BRD4	Bromodomain-containing Protein 4
cDNA	Complementary DNA
CO_2_	Carbon Dioxide
CRPC	Castration-Resistant Prostate Cancer
Ct/Cq	Threshold Cycle/Quantification Cycle
CXCR3	C-X-C Motif Chemokine Receptor 3
CXCR7	C-X-C Motif Chemokine Receptor 7
DMSO	Dimethyl Sulfoxide
DMEM	Dulbecco’s Modified Eagle Medium
DNA	Deoxyribonucleic Acid
EMT	Epithelial–Mesenchymal Transition
FBS	Fetal Bovine Serum
HEK-293	Human Embryonic Kidney 293 Cells
HOXB13	Homeobox B13
IC_50_	Half-Maximal Inhibitory Concentration
IC_25_	25% Inhibitory Concentration
IL-6	Interleukin-6
IL-6R	Interleukin-6 Receptor
MAPK	Mitogen-Activated Protein Kinase
miRNA/miR	MicroRNA
mRNA	Messenger RNA
mTOR	Mechanistic Target of Rapamycin
MTT	3-(4,5-Dimethylthiazol-2-yl)-2,5-diphenyltetrazolium bromide
NF-κB	Nuclear Factor Kappa-light-chain-enhancer of Activated B Cells
PBS	Phosphate-Buffered Saline
PC3	Prostate Cancer Cell Line 3
PI3K	Phosphoinositide 3-Kinase
PTEN	Phosphatase and Tensin Homolog
qPCR/RT-qPCR	Quantitative Polymerase Chain Reaction/Reverse Transcription Quantitative PCR
RISC	RNA-Induced Silencing Complex
RNA	Ribonucleic Acid
RNA-seq	RNA Sequencing
ROS	Reactive Oxygen Species
RPMI	Roswell Park Memorial Institute Medium
SD	Standard Deviation
SE/SEM	Standard Error of the Mean
STAT3	Signal Transducer and Activator of Transcription 3
TGF-β	Transforming Growth Factor Beta
TP53	Tumor Protein 53 (p53)
U6	U6 Small Nuclear RNA (Endogenous Control)
